# Design of an On-Chip Plasmonic Modulator Based on Hybrid Orthogonal Junctions Using Vanadium Dioxide

**DOI:** 10.3390/nano11102507

**Published:** 2021-09-26

**Authors:** Gregory Beti Tanyi, Miao Sun, Christina Lim, Ranjith Rajasekharan Unnithan

**Affiliations:** Department of Electrical and Electronic Engineering, Faculty of Engineering and Information Technology, The University of Melbourne, Melbourne, VIC 3010, Australia; aussunnysun@gmail.com (M.S.); chrislim@unimelb.edu.au (C.L.)

**Keywords:** electro-optics, modulators, nanophotonics, phase-changing materials, plasmonics

## Abstract

We present the design of a plasmonic modulator based on hybrid orthogonal silver junctions using vanadium dioxide as the modulating material on a silicon-on-insulator. The modulator has an ultra-compact footprint of 1.8 μm × 1 μm with a 100 nm × 100 nm modulating section based on the hybrid orthogonal geometry. The modulator takes advantage of the large change in the refractive index of vanadium dioxide during its phase transition to achieve a high modulation depth of 46.89 dB/μm. The simulated device has potential applications in the development of next generation high frequency photonic modulators for optical communications which require nanometer scale footprints, large modulation depth and small insertion losses.

## 1. Introduction

Advances in nanofabrication in the past 30 years have led to the development of more compact and faster photonic and electro-optic devices [1,2,3,4]. Modulators are electro-optic devices that encode a high-speed electronic data stream to an optical carrier wave in photonic integrated circuits for optical communications. The size, power consumption and the frequency of operation are the key metrics used to evaluate performances of the modulators. Recently, there has been a surge in the development of novel modulators based on silicon photonics due to its compatibility with complementary metal-oxide-semiconductor (CMOS) technology [4]. This compatibility enables the integration of photonic circuits and electronic circuits in a single chip to increase the speed of operation and to reduce footprint [5].

However, reducing the footprint while keeping the speed of operation is still an on-going challenge. This is because the footprints of conventional silicon photonics optical devices are limited by the diffraction limit. Also, silicon does not exhibit a linear electro-optic (EO) effect and thus silicon photonic modulators operate either on the dispersion effect or by integrating silicon with materials which exhibit an EO effect [5]. Silicon modulators based on the dispersion effect operate by varying the carrier concentration which in turn tunes the permittivity of silicon. This approach leads to limitations in the modulation depth, size and power requirements of the devices. The other approach which involves integrating silicon with EO materials requires a large interaction length (footprint) between the electrical signals and the optical signals.

To circumvent these constraints, plasmonic- based devices (surface plasmon polaritons (SPPs)) have been integrated in silicon photonics [6]. SPPs are electromagnetic surface waves at a dielectric–metal interface, coupled to the charge density oscillation in the metal surface [5,6]. SPPs offer the ability to focus light on nanoscales and are key elements in the development of subwavelength optical components with the added advantage of being compact and operating at much higher frequencies [1,6,7]. Recently, Graphene based plasmonic modulators have been used to achieve a modulation efficiency of 0.417 dB/µm [8]. Also, plasmonic modulators based on the Pockels effect and operating at 40 GHz have been developed using electro-optical polymers with a footprint of 29 µm [9]. Designing plasmonic devices usually entails a trade-off between modulation depth, device size, loss and extinction ratio [9,10]. There is a demand for plasmonic modulators with small footprints, as well as low radiative and dissipative losses [5,6,7,9].

Vanadium dioxide (VO_2_) is a canonical Mott material which exhibits a first order insulator to metal transition (IMT) which can be triggered by exciting the material thermally, electrically, or optically [10,11]. This phase transition is accompanied by a corresponding large change in the refractive index in the bulk material. It has been experimentally shown that an electric field strength of 6.5 × 10^7^ V/m would trigger the insulator to metal transition of VO_2_ [12,13]. This phase transition occurs at the femto second scale (26 fs) and as such, VO_2_ has been used as the modulating material in many high speed plasmonic devices. In [10], a thermally driven switch based on VO_2_ has been developed with an extinction ratio of 6.4 dB/μm and a 5 μm modulation section. However, thermally driven VO_2_ devices have a limited speed of operation. In [13,14,15,16,17,18,19], several VO_2_ based plasmonic modulators are explored. Some of these devices are limited by their footprint, while others require initial heating to trigger the transition due to the large size of the modulation section, and yet others have relatively low modulation depths.

In this paper, we report the simulation of a hybrid orthogonal plasmonic modulator with a 100 nm × 100 nm modulation section within a compact device footprint of 1.8µm × 1.0 µm. The electro-optic material of choice is vanadium dioxide due to the large change in the refractive index with the semiconductor phase having a refractive index of 3.24 + 0.30i and the metallic phase having a refractive index of 2.03 + 2.64i [12]. The optical modulation in this device is achieved by the large refractive index change of the nanoscale VO_2_ in the plasmonic slot under an external electric field. The orthogonal coupling geometry makes the device footprint small and provides a high modulation index of 46.89 dB/µm at the telecommunication wavelength, 1550 nm. Furthermore, the orthogonal geometry parameters are investigated and optimized with an in-depth simulation study, showing the device has a broad operating wavelength.

The rest of this paper is organized as follows: In Section 2, we present the general modulator design and its operation principle. In Section 3, we provide a discussion of the simulation methodology, perform an in-depth study on the performance of the device when tuning its geometry across a wide wavelength range and interpret the results obtained. Finally, a conclusion and brief discussion on areas of application of the device is given in Section 4.

## 2. Materials and Methods

**Figure 1:**
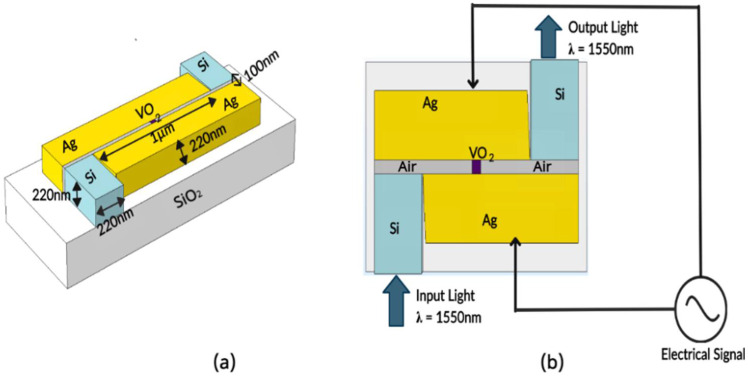
(**a**) Three-dimensional geometry of the proposed hybrid orthogonal plasmonic modulator. (**b**) Two-dimensional geometry of the proposed hybrid orthogonal plasmonic modulator showing light coupling directions. (Top layer of air not shown for visibility).

The design of this modulator achieved with the following goals: maximizing the modulation depth and reducing the physical dimensions of the device, as well as minimizing the power consumption of the device while maintaining a broad wavelength of operation. The proposed modulator geometry is shown in Figure 1. The coupling scheme used is similar to the published work in [20] based on a metal-insulator-metal (MIM) plasmonic waveguide.

In this geometry, the light of wavelength 1550 nm travels in a silicon waveguide (width 430 nm and height 220 nm) and is then coupled at the orthogonal silicon-air junction to a plasmonic slot waveguide (100 nm width and 1 μm length). The plasmonic slot is made of 220 nm thick silver due to its relatively low plasmonic losses [21]. A 100 nm long section of VO_2_ is introduced at the center of the plasmonic slot as the modulating section. The silver electrodes can be extended via wire bonding and connected to an external voltage source to apply voltage across the slot to change the phase of VO_2_. At the second orthogonal junction, the plasmons are coupled back into photons and travel along the output silicon waveguide. A grating coupling scheme (not shown in the figure above) is used for coupling light in and out of the modulator.

The device operates in 2 states depending on the application of an external electric field which drives the insulator to metal transition of the VO_2_ in the modulating section with. The refractive index of VO_2_ in both states is obtained from literature [12,13]. In the OFF state, there is no external electric field applied and hence VO_2_ in the modulating section is in the semiconductor phase with a refractive index of (3.24 + 0.30i). In this phase, surface plasmon polaritons travel across the modulating section and interact with VO_2_ in its semiconductor phase. In the ON state, there is an electric field strong enough to trigger the semiconductor to metal transition (IMT) of VO_2_. In the metallic phase, VO_2_ has a lower refractive index but higher extinction coefficient (2.03 + 2.64i) and which leads to the surface plasmon polaritons being attenuated by the VO_2_ section. The surface plasmon propagating in the slot through VO_2_ get modulated as it toggles between the ON and OFF states. The modulation depth is described as how much the modulation variable of a propagating carrier varies around its normal unmodulated level. Here, the modulation variable is the optical loss (attenuation). The optical loss is measured as the ratio of output power to the input power which are obtained by calculating the surface integral of the optical power at the silicon waveguides (input port and output ports) [22].

In the orthogonal modulator, the silicon waveguide has a width of 430 nm and a height of 220 nm, the angular separation (θ) between the silicon waveguide and the silver electrode is 10°, the width of the plasmonic slot (w) is 100 nm and the length of VO_2_ (L) used is 100 nm. Silver electrodes of height 220 nm are used as walls of the plasmonic slot, and the middle of the slot is filled with a 100 nm long section of VO_2._ The above parameters are optimized for operation in the C-band frequency range as shown in Figure 1b. The simulation details used to obtain the optimal parameters for the modulator along with the results are discussed in the following section.

## 3. Simulation Results and Discussion

We perform the modulator design, simulation and optimization using the finite element methods implemented in COMSOL Multiphysics commercial software (Version 5.2, COMSOL AB, Stockholm, Sweden). A minimum mesh element size of 6 nm is used in the plasmonic slot section which contains the smallest device features. Scattering boundary conditions (SBCs) are utilized to terminate the computational domain. The refractive indices of VO_2_ were obtained from literature as measured with the aid of variable angle spectroscopic ellipsometry as presented in [12,13]. Table 1 shows the material refractive index at the telecom wavelength (1550 nm).

**Figure 2:**
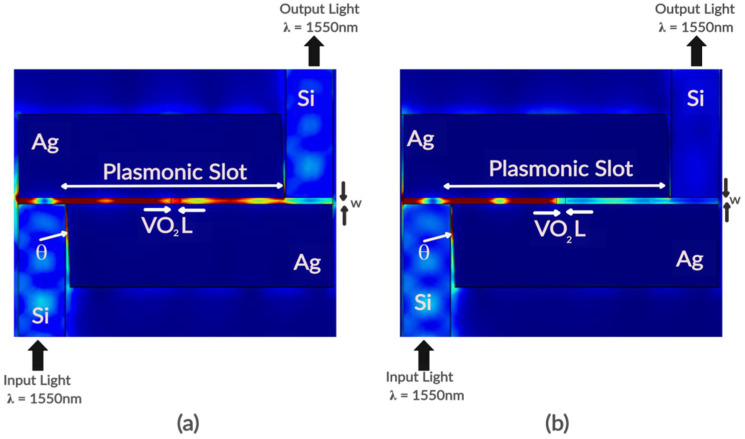
Planar cross-section view of device showing surface plot of electric field norm in (**a**) Left Semiconductor phase of VO_2_ (Device OFF) and (**b**) Right Metallic phase (Device ON) of VO_2_.

Figure 2a shows a top view of the modulator in the OFF state. In this state, light in the silicon waveguide is coupled to the plasmonic slot and then propagates as SSPs through the slot interacting with VO_2_ in the semiconductor phase before being coupled back into light at the output waveguide. Figure 2b shows the same top view of the device in the ON state. In the ON state, the metallic VO_2_ attenuates the travelling plasmons significantly, hence reducing the intensity of light coupled back at the output waveguide.

**Figure 3:**
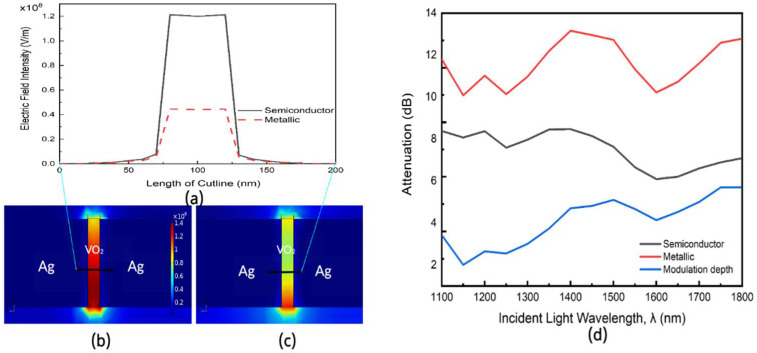
(**a**) Electric field Norm variation along cutline through plasmonic slot showing strong field confinement, Longitudinal cross section of the device in (**b**) OFF state (VO_2_ in semiconductor phase) (**c**) ON state (VO_2_ in metallic phase) (**d**) Insertion loss in both the metallic phase (ON phase) and the semiconductor phase (OFF phase) for incident light wavelength (λ) swept from 1100 nm to 1800 nm from simulation study.

Figure 3b,c show a cross section of the electric field across the Ag-VO_2_-Ag plasmonic slot with a strong confinement of the electric field within the slot. It is observed that in the ON state of the device, the electric field intensity of plasmons is lower than in the OFF state because of the higher optical loss which accompanies the phase change of VO_2_ to the metallic phase. We further examine the electric field confinement in both states by examining the electric field intensity along a cutline in the modulating section of the plasmonic slot as shown in Figure 3a. To obtain the optical attenuation, we first calculate the ratio of the surface integral of the optical power Poynting vector (S) at both ends of the silicon waveguide which are the input and output ports [21,22]. The Poynting vector is obtained as

(1) $$S\left[W∕m^{2}\right]=\;\hat{n}I$$

The surface integral of the optical Poynting vector at the input and output ports give us the input power (*P_input_*) and output power (*P_output_*), respectively. The optical power (*P*) and optical attenuation are given by the equations below:

(2) $$P=\;\iint \;\vec{S}\;\cdot \;\hat{n}\;\mathrm{da}\;\mathrm{Attenuation}\;\left(\mathrm{dB}\right)=-10\log\left(\frac{P_{\mathrm{output}}}{P_{\mathrm{input}}}\right)$$

The modulation depth (dB) is given by the difference in attenuation of the device in the OFF and ON states. It can be seen from Figure 3a that there is a significant drop in electric field intensity of the plasmons, which is consistent with Figure 2 and results from the interaction of the plasmons with VO_2_ in its metallic phase with a high extinction coefficient.

Figure 3d shows the modulation depth of the device in both states along a range of wavelengths (1100–1800 nm) in the telecommunication. The device is optimized to have a minimal insertion loss around the C Band of the telecommunications window.

**Figure 4:**
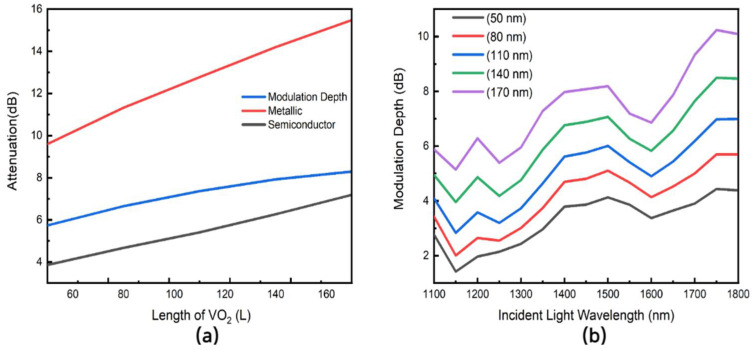
(**a**) Attenuation of light of wavelength 1550 nm for both the metallic and semiconductor phases of VO_2_ as well as the modulation depth for different lengths of the VO_2_ modulating section (L). (**b**) The variation of the modulation depth with different wavelengths of incident light (λ) from 1100 nm to 1800 nm, with the length of the VO_2_ modulating section (L) varied from 50 nm to 170 nm.

A key parameter that affects the level of modulation is the length (L) of VO_2_ used in the device slot. In the simulations, the length (L) was varied from 50 nm to 170 nm at the 1550 nm wavelength while keeping the other parameters constant. These values were chosen because of the limitation of modern nanofabrication techniques and are well within the decay length of the plasmons. Figure 4a shows how the attenuation and modulation depth vary with the length of VO_2_ at 1550 nm wavelength. We observe a linear increase in attenuation as the length of VO_2_ in the slot is increased. This can be explained by the extinction coefficient of VO_2_ in both phases. Given VO_2_ is not a transparent material, the plasmons are attenuated when they interact with the modulating section and as such, increasing the length of VO_2_ means more interaction and hence loss in the plasmon energy. The higher loss in the metallic phase is because of a higher extinction coefficient in the metallic phase.

We also study the effect of varying the wavelength on the device performance. For this, we perform a wavelength sweep from 1100 nm to 1800 nm with the length (L) of VO_2_ varied from 50 nm to 170 nm and compare the attenuation in both the metallic and semiconductor phases. The results in Figure 4b show that the device is robust enough to handle wavelength shifts due to transient temperature effects in high power laser systems. The trend of increased attenuation with an increase in length of VO_2_ is consistent in both Figure 4a,b. The slight dips at wavelengths 1150 nm and 1600 nm wavelengths are from the refractive index data used obtained from literature [12,13].

**Figure 5:**
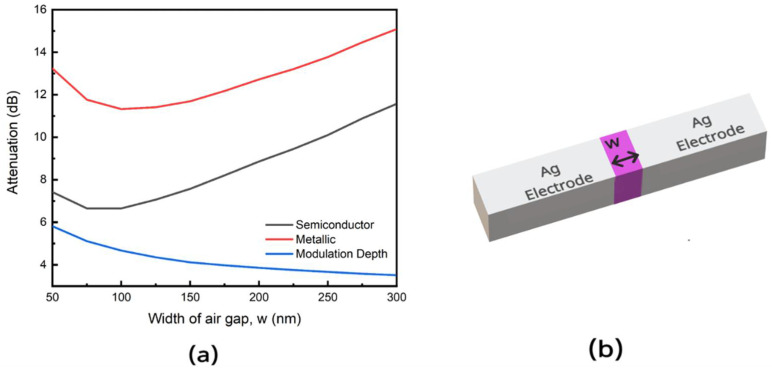
(**a**) Attenuation of light at 1550 nm for the metallic and semiconductor phases of VO_2_ with the modulation depth for different widths w of the plasmonic slot. (**b**) Cross section of the plasmonic slot of width (w) with violet colour representing VO_2_ and yellow colour representing Ag electrode.

In the fabrication of the device, there is a need to understand the impact of the width of the plasmonic slot (w) on the device performance, as well as the angle separating the electrodes from the waveguides (θ) given these constitute the critical dimensions of the device and are most susceptible to slight variations given the limitations of the current nanofabrication technology [23,24,25,26,27,28,29,30]. We thus study the impact of increasing the width of the plasmonic slot (w) on the attenuation in both phases and the modulation depth. Figure 5a shows a decrease in modulation depth as the width is increased. This is because increasing the width of the plasmonic slot reduces the photon to plasmon coupling efficiency.

The dependence of the modulation depth of the device on the angular separation is also studied by sweeping the angular separation (θ) from 0–80°. From the simulation results, we observe that the modulation depth reduces as the angular separation is increased. This is because a smaller angular separation accounts for a higher coupling efficiency.

**Figure 6:**
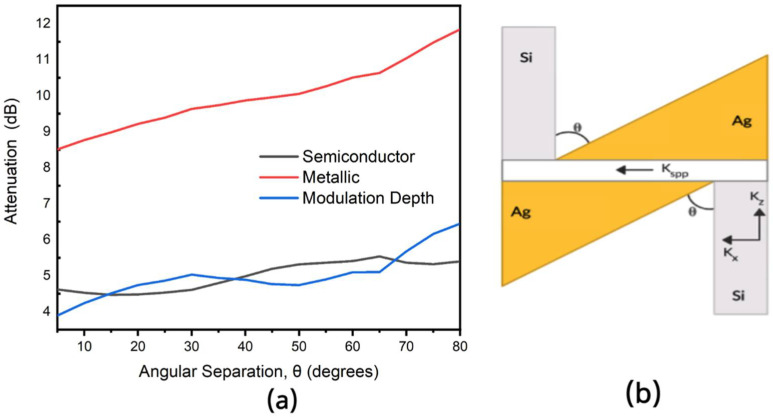
(**a**) Attenuation of Light at 1550 nm for the metallic and semiconductor phases of VO_2_ with the modulation depth for different angular separations (θ) between the Ag electrode and the silicon nanowire. (**b**) 2D-schematics of device with Silver highlighted in yellow and silicon highlighted in grey along with transverse component of silicon nanowires momentum (k_x_), orthogonal component of silicon waveguide momentum (k_y_) and surface plasmon polariton momentum annotated (k_spp_).

**Figure 7:**
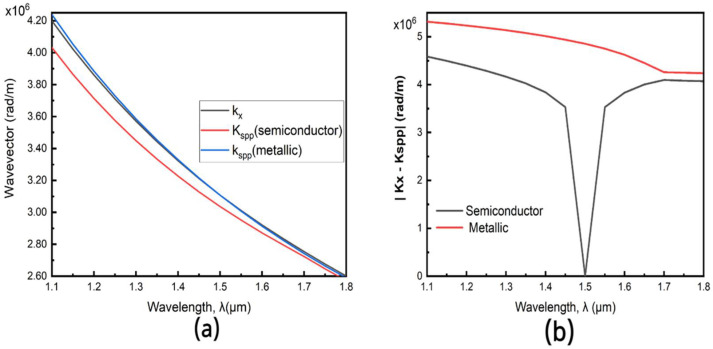
(**a**) Dispersion characteristics of the plasmonic slot and the silicon waveguide with VO_2_ in both the metallic and semiconductor phases. The slot width (w) is 100 nm, the silicon waveguide is 430 nm wide and 220 nm high (**b**) Absolute value of spatial mismatch in the metallic (ON) and metallic (OFF).

Using only a 100 nm modulation section of VO_2_ and with the following optimal parameters (w = 100 nm, θ = 8°), we achieve a modulation depth of 4.69 dB (46.89 dB/μm). From Figure 2 and Figure 3, a high extinction coefficient plays a key role in achieving such high modulation depth. We further investigate the effect of the phase change of VO_2_ on the coupling efficiency of this hybrid orthogonal geometry. In this hybrid-orthogonal geometry, the coupling efficiency is highly related to the momentum mismatch between the SPP mode and the waveguide [9,23]. Maximum coupling occurs when the orthogonal component of the momentum of the waveguide (k_x_) matches the fundamental mode of the plasmonic slot (K_spp_) and there is thus a minimal spatial mismatch. The schematics of this coupling scheme is illustrated in Figure 6b. In Figure 7, the longitudinal wave vector (and thus momentum) of the plasmonic slot with VO_2_ in both the metallic phase and semiconductor phase is compared to the orthogonal component of the waveguide’s wave vector (and thus momentum). Figure 7 shows that at a wavelength of 1550 nm, the plasmonic slot with VO_2_ in the semiconductor phase K_spp_ equals K_x_. Also, observing both curves shows the device has a higher coupling efficiency with VO_2_ in the semiconductor phase than the metallic phase, as the device was optimized for the semiconductor phase. This change in the coupling efficiency, as well as the high extinction ratio of VO_2_ lead to the high modulation depth as shown.

## 4. Conclusions

In this paper, we have demonstrated a hybrid orthogonal plasmonic modulator with a compact 100 nm × 100 nm modulating section and a device footprint of 1.8 μm × 1 μm. The device uses Vanadium dioxide (VO_2_) as the electrooptic material for intensity modulation and exploits the large change in the refractive index of VO_2_. The modulator is optimized for the 1550 nm telecommunications wavelength but can operate broadly in the O, E, S, C, L and U Bands (1260–1675 nm) and outperforms similar devices in literature to achieve a modulation depth of 46.89 dB/μm at the 1550 nm. The results will have applications in design of compact, cutting-edge high frequency modulators for high-speed optical communications.

## Figures and Tables

**Table 1:** Material Refractive Index (1550 nm).

VO_2_ (Semiconductor)	VO_2_ (Metallic)	Si	SiO_2_
3.24 + 0.30i	2.03 + 2.64i	3.46	1.45

## References

[1] Rajasekharan R., Wilkinson T.D., Hands P.J.W., Dai Q. (2011). Nanophotonic Three-Dimensional Microscope. Nano Lett..

[2] Yong J., Hassan B., Liang Y., Ganesan K., Rajasekharan R., Evans R., Egan G., Kavehei O., Li J., Chana G. (2017). A Silk Fibroin Bio-Transient Solution Processable Memristor. Sci. Rep..

[3] Rajasekharan R., Dai Q., Wilkinson T.D. (2010). Electro-optic characteristics of a transparent nanophotonic device based on carbon nanotubes and liquid crystals. Appl. Opt..

[4] Chrostowski L., Hochberg M. (2016). Silicon Photonics Design.

[5] Jagadish C., Lourdudoss S., Bowers J.E. (2019). Future Directions in Silicon Photonics.

[6] Gramotnev D.K., Bozhevolnyi S. (2010). Plasmonics beyond the diffraction limit. Nat. Photon..

[7] Abate Y., Marvel R.E., Ziegler J.I., Gamage S., Javani M.H., Stockman M.I., Haglund R.F. (2015). Control of plasmonic nanoantennas by reversible metal-insulator transition. Sci. Rep..

[8] Zhou F., Liang C. (2019). Highly tunable and broadband graphene ring modulator. J. Nanophotonics.

[9] Heni W., Haffner C., Elder D.L., Tillack A.F., Fedoryshyn Y., Cottier R., Salamin Y., Hoessbacher C., Koch U., Cheng B. (2017). Nonlinearities of organic electro-optic materials in nanoscale slots and implications for the optimum modulator design. Opt. Express.

[10] Olivares I., Sánchez L., Parra J., Larrea R., Griol A., Menghini M., Homm P., Jang L.W., van Bilzen B., Seo J.W. (2018). Optical switching in hybrid VO_2_/Si waveguides thermally triggered by lateral microheaters. Opt. Express.

[11] Jager M.F., Ott C., Kraus P.M., Kaplan C.J., Pouse W., Marvel R.E., Haglund R.F., Neumark D.M., Leone S.R. (2017). Tracking the insulator-to-metal phase transition in VO_2_ with few-femtosecond extreme UV transient absorption spectroscopy. Proc. Natl. Acad. Sci. USA.

[12] Verleur H., Barker A., Berglund C. (1968). Optical Properties of VO_2_ between 0.25 and 5 eV. Phys. Rev..

[13] Earl S.K., James T.D., Davis T., McCallum J., Marvel R.E., Haglund R.F., Roberts A. (2013). Tunable optical antennas enabled by the phase transition in vanadium dioxide. Opt. Express.

[14] Sun H., Zhao L., Dai J., Liang Y., Guo J., Meng H., Liu H., Dai Q., Wei Z. (2020). Broadband Filter and Adjustable Extinction Ratio Modulator Based on Metal-Graphene Hybrid Metamaterials. Nanomaterials.

[15] Markov P., Appavoo K., Haglund R.F., Weiss S.M. (2015). Hybrid Si-VO_2_-Au optical modulator based on near-field plasmonic coupling. Opt. Express.

[16] Sun M., Shieh W., Unnithan R.R. (2017). Design of Plasmonic Modulators with Vanadium Dioxide on Silicon-on-Insulator. IEEE Photonics J..

[17] Kim Y., Wu P.C., Sokhoyan R., Mauser K.A., Glaudell R., Shirmanesh G.K., Atwater H.A. (2019). Phase Modulation with Electrically Tunable Vanadium Dioxide Phase-Change Metasurfaces. Nano Lett..

[18] Olyaee M., Tavakoli M.B., Mokhtari A. (2018). Analyze and calculation of coupling coefficient based on evanescence field for plasmonic directional coupler structure. Opt. Quantum Electron..

[19] Markov P., Marvel R.E., Conley H.J., Miller K.J., Haglund J.R.F., Weiss S.M. (2015). Optically Monitored Electrical Switching in VO_2_. ACS Photon..

[20] Lau B., Swillam M.A., Helmy A.S. (2010). Hybrid orthogonal junctions: Wideband plasmonic slot-silicon waveguide couplers. Opt. Express.

[21] Zouhdi S., Sihvola A. (2009). Metamaterials and Plasmonics: Fundamentals, Modelling, Applications.

[22] Cheng D. (2014). Field and Wave Electromagnetics.

[23] Noghani M.T., Samiei M.H.V. (2014). Propagation Characteristics of Multilayer Hybrid Insulator-Metal-Insulator and Metal-Insulator-Metal Plasmonic Waveguides. Adv. Electromagn..

[24] Kruger B.A., Joushaghani A., Poon J.K.S. (2012). Design of electrically driven hybrid vanadium dioxide (VO_2_) plasmonic switches. Opt. Express.

[25] Rajasekharan R., Butt H., Dai Q., Wilkinson T.D., Amaratunga G.A.J. (2012). Can Nanotubes Make a Lens Array?. Adv. Mater..

[26] Wu B., Zimmers A., Aubin H., Ghosh R., Liu Y., Lopez R. (2011). Electric-field-driven phase transition in vanadium dioxide. Phys. Rev. B.

[27] Zhang C., Wang D., Huang S., Yang J., Liu J., Fang J. (2021). Nonlinear Optical Response of Gold Nanobipyramids for a Doubly Q-Switched Ho-Doped Laser at a Wavelength of 2.1 µm. Nanomaterials.

[28] Sopko I.M., Knyazev G.A. (2016). Optical modulator based on acousto-plasmonic coupling. Phys. Wave Phenom..

[29] Salamin Y., Baeuerle B., Heni W., Abrecht F.C., Josten A., Fedoryshyn Y., Haffner C., Bonjour R., Watanabe T., Burla M. (2018). Author Correction: Microwave plasmonic mixer in a transparent fibre–wireless link. Nat. Photon..

[30] Xu X., Chung C.-J., Pan Z., Yan H., Chen R.T. (2018). Periodic waveguide structures for on-chip modulation and sensing. Jpn. J. Appl. Phys..

